# Self-Blood Pressure Monitoring (SBPM) in Patients With Hypertension and Multimorbidity: A Systematic Review

**DOI:** 10.7759/cureus.77160

**Published:** 2025-01-08

**Authors:** Taiwo A Falaiye, Okelue E Okobi, Christiana U Ndoh, Chioma C Ubajaka

**Affiliations:** 1 Family Medicine, Northwest Health - La Porte, La Porte, USA; 2 Family Medicine, Medficient Health Systems, Laurel, USA; 3 Family Medicine, Lakeside Medical Center, Belle Glade, USA; 4 Family Medicine, Larkin Community Hospital Palm Springs Campus, Hialeah, USA; 5 Family Medicine, Garki Hospital Abuja, Abuja, NGA; 6 Internal Medicine, Igbinedion University Okada, Benin City, NGA

**Keywords:** blood pressure, blood pressure control, chronic disease management, hypertension, multimorbidity, self-blood pressure monitoring

## Abstract

Hypertension (HTN) remains a major cause of mortality and morbidity globally and is normally accompanied by multimorbidity, which makes its management increasingly difficult and multifaceted. In addition to being considered a vital intervention empowering patients by ensuring they are actively involved in their care and enhancing HTN management, several studies have disclosed that self-blood pressure monitoring (SBPM) is highly effective when utilized jointly with other interventions. However, the effectiveness of SBPM varies in instances of multimorbidity. This systematic review aims to evaluate the effectiveness of SBPM in reducing clinic BP in persons with HTN and multimorbidity. To realize the study objectives, an in-depth literature search and systematic review were performed on articles drawn from various virtual databases, including PubMed, SCOPUS, Medline, Google Scholar, and Embase. Randomized controlled trials (RCTs), cohort studies, clinical trials, and reviews focused on SBPM were selected and included. As a result, 12 studies met the inclusion criteria and were included based on the Preferred Reporting Items for Systematic Reviews and Meta-Analyses (PRISMA) guidelines. As such, SBPM was found to be effective in lowering BP regardless of the existence of HTN-related comorbidities; however, SBPM was only effective in instances of comorbidities that included stroke and obesity and upon combination with other high-intensity co-interventions.

## Introduction and background

Hypertension (HTN) refers to an elevated blood pressure (BP) with a systolic blood pressure (SBP) that ranges between 120 and 129 mmHg, as well as a diastolic blood pressure (DBP) of below 80 mmHg [1-4]. In this regard, HTN has been classified into three distinct categories (mild, moderate, and severe) based on the levels of BP, to offer a clearer perspective on the severity of the condition. For instance, mild/stage 1 HTN refers to an SBP of 130 mmHg and 139 mmHg, and DBP between 80 mmHg and 89 mmHg, indicating early HTN. Moderate/stage 2 HTN refers to an SBP of between 140 mmHg and 159 mmHg and a DBP of between 90 mmHg and 99 mmHg, while severe/stage 3 HTN refers to an SBP of ≥160 mmHg and a DBP: ≥100 mmHg [1-4]. At present, HTN remains a chronic condition and a global public health concern as it affects nearly 1.28 billion individuals globally and has been acknowledged to significantly contribute to cardiovascular diseases (CVDs), premature mortality, and kidney failure in instances of poor management [1]. Thus, HTN is among the most common conditions in individuals with multimorbidity [1,2]. In this regard, multimorbidity refers to having two or more concomitant diseases, which affect approximately 10-50% of persons with HTN, depending on the population under study [1,3,4], and includes conditions such as coronary heart disease (CHD), chronic kidney disease (CKD), stroke (ischemic attack), diabetes, and obesity, among others. Multimorbidity has been linked to reduced patient quality of life [1,5]. Owing to the intricacies of studying persons with multimorbidity, a limited number of studies have focused on interventions developed to enhance the outcomes in persons with multimorbidity [6,7].

Optimal BP management is the most effective means of preventing CVD and stroke [8]. In this regard, self-blood pressure monitoring (SBPM), alongside self-management of BP, is considered effective in reducing BP in hypertensive patients [9]. Nevertheless, in persons with HTN and multimorbidity, it is feasible that such interventions might prove less effective as a result of clinical inertia on the treating physician’s part, as well as the patient’s concerns regarding SBPM, particularly in instances where co-morbidities exist [10-12]. Extant literature has not demonstrated the aptitude of SBPM to bring about enhancements in the management of risk factors in persons with HTN and multimorbidity [2,13], even as individual studies normally have a limited number of patients with multimorbidity to evaluate the findings with sufficient power, especially within subgroups. A recent study conducted to evaluate the effectiveness of SBPM, which included data drawn from 25 studies comprising 8931 patient participants, disclosed significant reductions in BP following the use of SBPM, with the efficacy of the tool being observed to increase with the increment in the co-interventions intensity level [14,15]. However, it has also been noted that in certain HTN patients with multimorbidity that include CVDs such as myocardial infarction and stroke, the observed enhancement of BP with the co-intervention intensity levels might significantly reduce [14]. To ensure a better comprehension of the effects of SBPM in persons with HTN and multimorbidity, the present study seeks to systematically evaluate the findings of various recent and previous studies to establish how SBPM affects patient care outcomes in such patients. Thus, this study aims to assess the effectiveness of SBPM in managing persons with HTN and multimorbidity by determining how the approach/tool affects the control of BP, patient adherence, and general care outcomes. However, unlike previous studies, this systematic review will also evaluate the effects of the intensities of various co-interventions on SBPM in persons with HTN and multimorbidity. The study will additionally assess the extant barriers to SBPM usage in persons with HTN and multimorbidity and recommend practical solutions for optimum use within the clinical practice context.

## Review

Materials and methods

For this systematic review, we conducted an in-depth literature search on various virtual databases, including PubMed, Embase, Web of Science, SCOPUS, and Google Scholar search engine. The selected articles included epidemiological and health assessment studies comprising anonymized data alongside multi-center studies and published review articles. A comparison of the selected studies and articles from the same population years was conducted to identify duplicate data sources, leading to the selection and utilization of only studies with increasingly valid details.

Literature Search Strategy

The literature search strategy for pertinent studies involved an in-depth search of various virtual databases, including Google Scholar, PubMed, Embase, Web of Science, and SCOPUS. The search strategy included an amalgamation of Medical Subject Headings (MeSH) keywords that included “hypertension,” “blood pressure,” “self-blood pressure monitoring,” “multi-morbidity,” and “self-care,” using Boolean connecter “AND,” “NOT,” and “OR.” The in-depth literature search yielded a total of 1242 articles.

Inclusion and Exclusion Criteria

The inclusion criteria included randomized controlled trials (RCTs), prospective cohort studies, systematic reviews, and crossover design studies that met the following set criteria: studies that focused on HTN management and treatment, studies on HTN and multimorbidity, published in the English language, and conducted in the last 15 years. Consequently, sponsored clinical trials, editorials, opinion pieces, narrative reviews, dissertations, studies published in non-peer-reviewed journals, and studies initially published in non-English languages were excluded. Further, the extraction of vital data from the eligible pertinent studies was conducted as follows: (a) general attributes of the study, which included the names of the authors, year of study and publication, and the type of sampling methods used; (b) the characteristics of the study population, including sample size, race/ethnicity, age and gender of study participants, and follow-up; (c) type of intervention and duration; and (d) the main study findings.

Results

**Figure 1:**
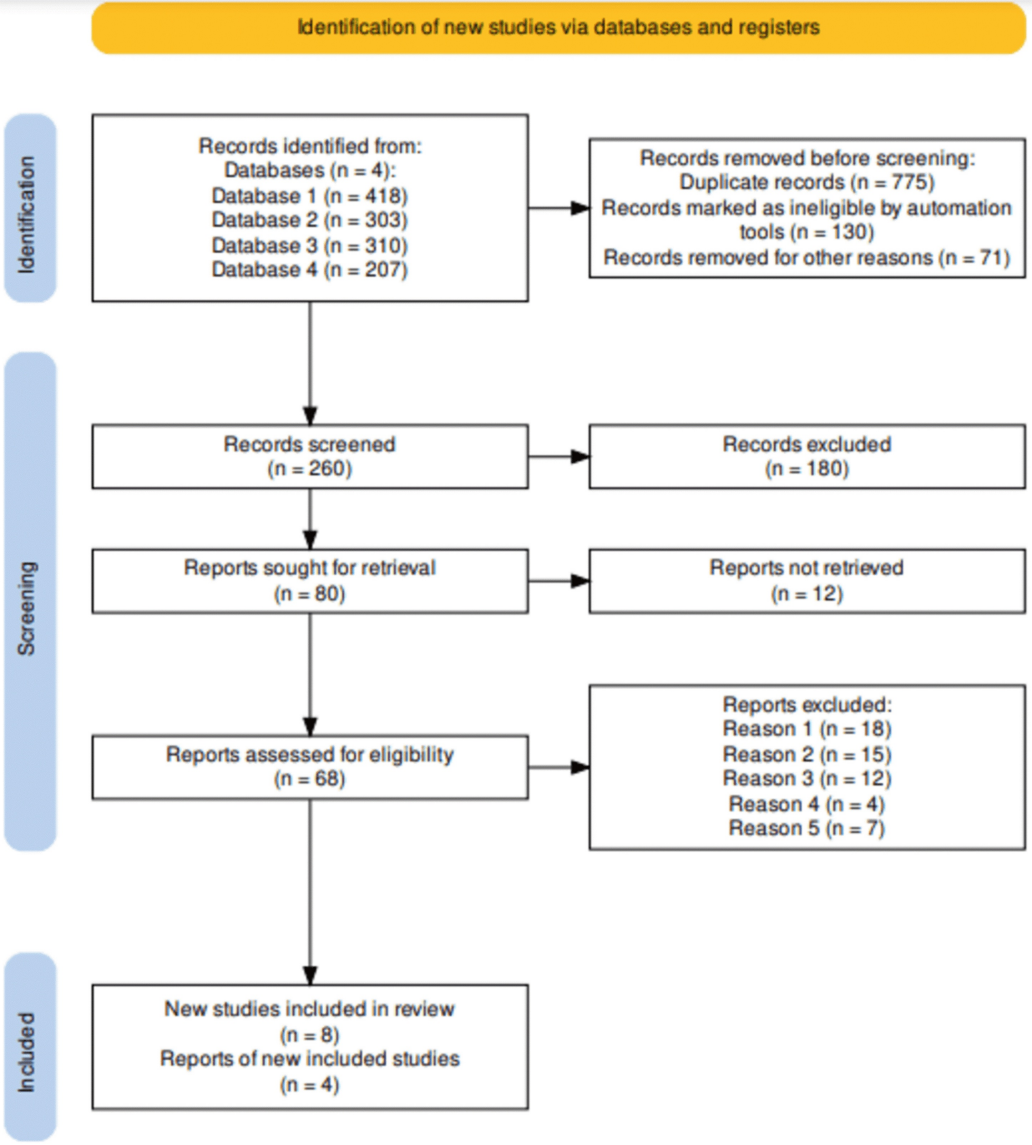
PRISMA flowchart indicating the study selection process for this systematic review Reasons 1-5 Reason 1: Irrelevant to the research question, aims and objectives Reason 2: Protocol issues Reason 3: Irretrievable full-text Reason 4: Failure to report the study limitations Reason 5: Published in non-peer-reviewed journal PRISMA: Preferred Reporting Items for Systematic Reviews and Meta-Analyses

For this systematic review, the study selection process was carried out based on the Preferred Reporting Items for Systematic Reviews and Meta-Analyses (PRISMA) guidelines. Therefore, the study selection resulted in the retrieval of 1238 article records following an in-depth search on virtual databases. The screening of the articles resulted in the removal of 775 duplicates even as another 130 studies were found ineligible through automation, and an extra 71 studies were excluded for other key reasons that included failure to align with the objectives of this study and animal-based studies. Therefore, 260 that met the inclusion criteria were screened, leading to a further exclusion of 180 studies. This led to the remaining 80 studies being sought for retrieval, with 12 articles being irretrievable. As such, 68 studies underwent further evaluation for eligibility, leading to the exclusion of 22 studies following full-text screening for reasons that included preprint (12 articles), protocol (18 articles), and full text missing even after reaching out to the authors (15 articles); failure to report limitations (four articles); and failure to investigate the targeted intervention (seven articles). Finally, 12 studies satisfied the set inclusion criteria and have been included in this systematic review, in addition to being assessed and discussed together with findings of other studies that have corroborated our study's findings [14-37]. The study selection process has been presented in the PRISMA flow diagram in Figure 1.

The summary of the studies included in this systematic review and their findings have been presented in Table 1.

**Table 1:** Summary of the included studies and their findings SBPM: Self-blood pressure monitoring; BP: Blood pressure; Med-STEP: Medication Self-titration Evaluation Program; COVID-19: Coronavirus disease 2019

Author Name/Year/Citation	Study Design	Sample Size	Population Attributes	Findings
Bryant et al. 2020 [14]	Retrospective cohort study	2590	Older patients who are hypertensive and are on SBPM	SBPM enhances hypertension care processes in addition to supporting long-term BP control. Moreover, SBPM interventions also improve treatment adherence and patient engagement.
McManus et al. 2010 [15]	Randomized controlled trial (RCT)	527	Patients aged between 35 and 85 years whose BP is over 140/90 mm Hg	Telemonitoring alongside SBPM results in improvement of BP control in hypertensive patients, in addition to demonstrating efficiency in the integration of technology with patient-driven care.
Wallace et al. 2015 [18]	Clinical review	319	Patients aged 70 years and above with multimorbidity	The study emphasized the need for tailored and well-coordinated primary care for BP patients with several chronic conditions. The findings additionally indicated that targeted interventions for multimorbidity are effective in improving health outcomes.
Muntner et al. 2020 [21]	RCT	348	Hypertensive patients	Home BP monitoring has been found to be cost-effective and is increasingly preferred by patients. The study has identified different barriers to clinical adoption of SBPM, including patient education and device ease of use.
Grant et al. 2012 [22]	Qualitative analysis of focus group	70 patients	Participants aged 40 to 65 years have a baseline blood pressure of over 139/84 mmHg	The study disclosed that Med-STEP, a virtual self-titration tool, is important in enhancing medication adherence and BP management. Further, it has been indicated that digital tools are effective in empowering patient self-management.
Hatef et al. 2022 [25]	Retrospective cohort study	1996347	Participants aged 18 years and above	The study compared in-person and telehealth outcomes during COVID-19 and disclosed that telehealth was effective and that there is a need to balance virtual and physical care.
Kaambwa et al. 2014 [26]	RCT	478 participants	Patients aged 35-85 years with baseline BP of over 140/90 mmHg, on treatment for hypertension, and willing to partake in SBPM	Self-monitoring with self-titration of antihypertensives and telemonitoring of blood pressure measurements not only reduces blood pressure, compared with usual care, but also represents a cost-effective use of health care resources.
Sheppard et al. 2020 [27]	Systematic review and meta-analysis	22 trial studies	Randomized controlled trials of SBPM alongside individual patient data (IPD)	SBPM is beneficial to hypertension patients with related multimorbidity. The study has also indicated variability in the results based on the individual patient data.
Tucker et al. 2015 [28]	Individual patient data meta-analysis	8931 patients	The population of patients with outpatient-managed hypertension and on SMBP interventions. RCTs with a minimum of 100 participants followed up for at least 24 weeks	The BP-SMART protocol aggregates data from various studies with regard to SBPM. The study has indicated that SBPM is increasingly effective in the control and management of BP.
Katon et al. 2010 [29]	RCT	214 patients	Hypertension patients with poorly controlled diabetes, coronary heart disease, or coexisting depression	The study has indicated that collaborative care models enhance the outcomes for depression alongside other chronic conditions, including hypertension. The study has further highlighted the significance of interdisciplinary approaches.
Kerry et al. 2013 [30]	RCT	381 participants	Patients attending outpatient and inpatient stroke clinics	The study has indicated that SBPM monitoring with nurse-led support enhances BP control in post-stroke patients. The study has demonstrated that community-based models for managing hypertension are very effective in BP management.
Volpi et al. 2021 [33]	Non-RCT	49 participants	Patients diagnosed with hypertension and are on medical treatment	Mobile health apps enhance patient adherence to hypertension treatment plans. Suggests digital platforms as effective tools for enhancing patient engagement and outcomes.

Risk of Bias (RoB) Assessment

For this systematic review, a RoB was conducted with the objective of evaluating the included studies’ internal validity. Thus, the objective of conducting RoB is to determine if the results and findings of the included studies have been affected by biases that might result in the under- or overestimation of the actual effects of the association and intervention. For this study, the Measurement Tool to Assess Systematic Reviews (AMSTAR) 2 critical appraisal tool was employed. Amongst the notable domains used in evaluating the quality of the studies are reporting bias, detection bias, selection bias, and confounding bias. The findings of the RoB assessment have been presented in Table 2.

**Table 2:** Risk of bias assessment table for this systematic review BP: Blood pressure; RCT: Randomized controlled trial

Study/Citation	Bias Domain	Judgement/ Risk Level	Support for Judgment
Bryant et al. [14]	Reporting bias	Low	The study has depended on objective blood pressure measurements, thereby minimizing the potential for detection bias.
	Detection bias	Low	The study has reported the primary and secondary outcomes, thereby minimizing the risk of reporting bias.
	Selection bias	Low	The study has described a sufficient randomization process thereby reducing the potential for allocation bias.
	Performance bias	High	Blinding of the study participants was not possible as a result of the intervention’s nature.
	Attrition bias	low	The reported attrition rates are low and aptly balanced across the study groups.
	Other bias	High	Potential funding influence details are limited.
McManus et al. [15]	Performance bias	Low	The blinding of the assessors aided in the reduction of potential risks.
	Reporting bias	Low	The study has fully reported the pre-specified outcomes.
	Selection bias	Low	The study is a randomized controlled trial and includes a clearer allocation concealment.
	Detection bias	Low	The BP measurements were objective.
	Attrition bias	Low	The missing data are minimal and have been aptly addressed.
	Other biases	Low	The study design is well-documented with no financial conflicts.
Wallace et al. [18]	Selection bias	Moderate	Owing to the study's observational design, the potential failure to carry out rigorous randomization might increase the possibility of selection bias.
	Performance bias	Low	Active patient and provider involvement intervention was included and this made blinding possible.
	Reporting bias	Low	The study has provided a clear report on the outcomes.
	Detection bias	Low	The study has provided sufficient details regarding the blinding of outcome assessors.
	Attrition bias	Low	Sensitivity analysis was used in addressing attrition.
	Other biases	High	Selection and publication biases from other studies.
Muntner et al. [21]	Confounding bias	High	The observational focus is likely to introduce the risk of confounding.
	Reporting bias	Moderate	Certain details regarding barriers, preferences, and study limitations might need to be included.
	Selection bias	Low	Clear randomization process and details provided
	Performance bias	Low	Active patient and provider involvement intervention was included and this made blinding possible.
	Detection bias	Low	Sufficient details regarding the blinding of outcome assessors were provided.
	Attrition bias	Low	Data completeness and sufficient handling of missing data reported.
	Other biases	High	Potential heterogeneity in the sources of data.
Grant et al. [22]	Selection bias	High	Given the study’s observational design, there is no randomization.
	Reporting bias	Low	The study has provided detailed implementation outcomes.
	Performance bias	High	Blinding was impossible owing to the intervention’s self-management nature.
	Detection bias	Low	The assessment of outcomes was objective and automated.
	Attrition bias	Moderate	The study reported a moderate dropout rate and offered limited discussion of how the dropout impacted the results.
	Other biases	Low	No major concerns, including funding conflicts, were reported.
Hatef et al. [25]	Performance bias	Low	Blinding was impossible for the telehealth and in-person interventions.
	Selection bias	High	The study’s observational design increased the potential confounders related to telehealth and in-person group attributes.
	Reporting bias	Low	More discussion is needed concerning the confounding factors.
	Detection bias	Low	depends on the analysis of telehealth outcomes.
	Attrition bias	Low	The study provided comprehensive data capture for all insured participants
	Other biases	Low	Robust data handling described
Kaambwa et al. [26]	Selection bias	Low	The study is based on the TASMINH2 trial, which entails a rigorous selection of participants.
	Reporting bias	Low	Every pertinent outcome, including cost-effectiveness, has been appropriately presented.
	Performance bias	High	The intervention type used significantly precluded participant blinding.
	Detection bias	Low	cost and health outcomes are objective
	Attrition bias	Low	Every outcome of interest reported.
	Other biases	Low	The study has offered comprehensive cost-efficiency methodologies.
Sheppard et al. [27]	Selection bias	Low	High-quality systematic review and meta-analysis that has broader inclusion criteria.
	Reporting bias	Low	The comprehensive reporting has reduced the risk of potential reporting bias.
	Performance bias	Moderate	The included studies might have lacked sufficient participant blinding.
	Detection bias	Low	The meta-analytical methodologies minimized potential individual study bias.
	Attrition bias	Low	Sufficient addressing of data from included studies was reported.
	Other biases	Low	No potential conflict of interest was reported.
Tucker et al. [28]	Performance bias	Low	The protocol stage, along with the transparent methodology, has minimized design bias.
	Reporting bias	Low	The study adheres to PRISMA guidelines, which have significantly lowered the risk of reporting bias.
	Selection bias	Low	A clear description of the randomization process is provided.
	Detection bias	Low	The study has reported independent and sufficient blinded data analysis.
	Attrition bias	Low	Clearer reporting and handling of participant dropout rates.
	Other biases	Low	The funding sources reported are unbiased.
Katon et al. [29]	Selection bias	Low	The study entails an RCT with robust methodologies.
	Performance bias	Moderate	Given that the blinding of clinicians is not achievable, there is a higher risk of performance bias.
	Detection bias	Low	Objective measurement of outcomes
	Attrition bias	Low	Sensitivity analysis was conducted and dropout rates were effectively balanced.
	Reporting bias	Low	The study reported every outcome specified in the protocol.
	Other bias	Low	Authorship details alongside the funding details suggested minimal bias.
Kerry et al. [30]	Performance bias	High	The intervention’s nature precluded participant blinding.
	Selection bias	Low	Sufficient randomization processes were described.
	Detection bias	Low	The assessment of study outcome was objective.
	Attrition bias	Low	The study presented a comprehensive reporting of all missing data with effective imputation methods.
	Reporting bias	Low	The study did not have any selective reporting concern.
	Other bias	Low	No significant bias sources reported.
Volpi et al. [33]	Performance bias	High	The non-randomized study design utilized increases bias risk.
	Selection bias	Low	The study design provided adequate allocation concealment.
	Detection bias	Moderate	Insufficient evidence on the blinding outcome assessor.
	Attrition bias	Moderate	The study provided inadequate information on dropout rates and the handling of missing data.
	Reporting bias	Low	The pre-registered protocol confirmed the appropriateness of outcome reporting.
	Other bias	High	Potential self-reported and confounding data concerns.

Quality Assessment

The quality of the studies included was assessed using the Appraisal Tool for Cross-Sectional Studies (AXIS) tool, which is a critical evaluation tool comprising 20 items. As such, three independent reviewers were tasked with evaluating the quality of each included study, and disagreements were mainly resolved through consensus and group discussions. Further, each included study was scored 1 (yes) or 0 (no), and “don’t know” for items inapplicable items, respectively. Overall, the included studies were of moderate to high quality, with only five studies being of moderate quality and the others being of high quality.

Data Extraction

A data extraction form was used by the authors to extract pertinent data from the included studies. Data on the different study attributes, including the publication year, authors’ names, research design, sample size, and study findings, were extracted from each study. The three independent reviewers were tasked with the extraction of the data, and potential disagreements were resolved through discussions and consensus.

Discussion

Normally, HTN is accompanied by multimorbidity, which comprises several chronic conditions that include CKD, diabetes, and CVDs, that results in difficulties for healthcare professionals tasked with navigation of the intricacies related to polypharmacy, customized treatment plans, and divergent disease trajectories [16]. It is approximated that 68% of adult persons with HTN in the United States have a minimum of one chronic comorbid condition, which contributes an additional layer of complexities that affects the treatment and management of the conditions [17]. According to Wallace et al., persons with HTN and multimorbidity always need specialized and individualized care from different healthcare professionals, which results in fragmentation, a dearth of effective coordination, and, eventually, suboptimal care outcomes [18]. Owing to the overlapping of different risk factors for HTN and other chronic conditions, along with their shared pathophysiological mechanism, an increasingly integrated management approach is needed. The conventional siloed healthcare delivery model, in which varied healthcare providers treat/manage each chronic condition in seclusion, has turned out to be insufficient in treating and managing persons with HTN and multimorbidity [19,20]. Rather, effective management of such patients requires a system that not only addresses such potential medical complexities but also takes into consideration the care delivery’s organizational aspects. Additionally, evidence drawn from the included studies in this systematic review suggests that SBPM can significantly improve the management of persons with HTN and multimorbidity. Amongst the notable advantages of SBPM use in persons with HTN and multimorbidity include the aptitude to enhance care coordination while simultaneously reducing fragmentation [14]. Notably, various studies have indicated that persons with HTN and multimorbidity receiving care via different kinds of system-based approaches often have fewer cases of hospital readmissions alongside reduced emergency department (ED) visits [2,14]. Through proper coordination of care by several providers, as well as the use of electronic health records (EHRs) in the documentation of the patient treatment progress, SBPM has been acknowledged to minimize instances of medication errors, duplicative testing, as well as potential inefficiencies occurring within fragmented care models.

Consequently, in relation to SBPM’s impact on BP control and management, it has been noted that the intervention aids in the reduction of BP over and above normal standard care through increased patient involvement in the treatment process. This, in turn, enhances the patient’s adherence while reducing clinical inertia and offering both the patient and healthcare experts information pertaining to the treatment’s effectiveness [1,21]. SBPM is also considered an effective indicator with regard to end-organ damages compared to the office or clinical assessment and has been well-tolerated by persons with HTN and multimorbidity [22].

Furthermore, SBPM has been acknowledged to facilitate the optimization of medication management, considered important for persons with HTN and multimorbidity and on increasingly intricate medications. For instance, Maher et al. maintain that the risk of adverse medication events is mainly increased by polypharmacy, which tends to worsen the main conditions or result in the patient developing novel comorbidities [23]. Further, by including various clinical decision-support tools and the use of interdisciplinary teams, SBPM facilitates the making of informed medication adjustment decisions by healthcare providers, thereby minimizing the probability of adverse drug interaction while enhancing the safety of the patient [23]. Additionally, through the incorporation of health information technologies, SBPM’s effectiveness is enhanced with regard to the management and treatment of persons with HTN and multimorbidity. Other tools, including decision-support systems and EHRs, facilitate the timely tracking of patient data, which enables healthcare professionals to aptly identify patterns, engage in proactive management of care, and adjust treatment interventions [24]. Further, telemedicine has turned out to be an important device within the framework of SBPM, as it enables remote monitoring of BP alongside other health parameters of persons with HTN and multimorbidity, thereby significantly reducing hospital visits while also enhancing access to care for the patients experiencing transportation and mobility challenges [25].

Regarding SBPM efficiency in the management of HTN with multimorbidity, the evaluation of the included studies reveals that SBPM has indicated improvements in the control and management of BP, particularly in instances where it was used alongside various co-interventions that included medication adjustment, telemonitoring, and nurse-led care [26-28]. A notable finding consists of the study conducted by McManus et al., who disclosed that the use of SBPM and the aptitude to receive consistent and timely feedback from healthcare professionals had significant positive effects as it lowered the patients’ SBP after 12 months in comparison to SBP of patient receiving normal care [26]. Similar findings have been disclosed in the study conducted by Sheppard et al., who observed that SBPM was linked to significant reductions in clinical SBP in comparison to normal care after a 12-month follow-up duration, irrespective of the total number of HTN-linked multimorbidity [27]. The study additionally disclosed that intense co-interventions were highly effective in comparison to the low-intensity interventions administered to obesity and CVD (stroke) patients [27]. Still, the study by McManus et al. that focused on persons with HTN and multimorbidity disclosed that, in the SBPM group, the mean SBP decreased by nearly 12.9mm Hg from the baseline to six months and that, in the control group, the reduction in SBP was 9.2 mm Hg during the same duration [15]. The study further disclosed that at 12 months, in the SBPM group, the SBP had reduced by 17.6 mm Hg, compared to 12.2 mm Hg in the control group [15]. Moreover, persons with HTN and various multimorbidities, including CKD, CVDs, and diabetes, have been acknowledged by multiple studies to have significantly gained from SBPM, given that it enabled them to detect BP fluctuations in real time, particularly in instances where the fluctuations were linked to medication adjustments and disease progressions [26]. However, regardless of the above observations, while Tucker et al. found SBPM to be related to reduced SBP in persons with HTN alongside obesity and diabetes, the study disclosed that there was an insignificant reduction of SBP in patients with CKD, CHD, and stroke [28]. Furthermore, a number of studies have further disclosed that SBPM was effective in aiding persons with HTN and heart failure by reducing the rate of hospital readmission by approximately 18% as a result of the timely identification of HTN worsening and apt interventions taken [14,27,28]. According to Sheppard et al., SBPM was linked to significant reductions in the probability of uncontrolled BP in persons with HTN and CKD, obesity, and diabetes at six-month follow-up, even though significant interactions were observed between the SBPM effects and intervention intensity in such patients, with patients offered high-intensity interventions being less prone to experiencing uncontrolled BP at six months [27].

In comparison to findings of studies that focused on the evaluation of ambulatory BP in persons with HTN and multimorbidity, and with no co-interventions, it was disclosed that there were no changes in ambulatory SBP linked to SBPM and ambulatory DBP [27,29]. Still, it is noteworthy that the effectiveness of SBPM in persons with HTN and multimorbidity has been a subject of intense debates, and a number of studies have maintained that the intervention is effective [27,29], while others have the efficacy of the intervention in relation to certain morbidities [30]. The present systematic review has corroborated the positive effects of SBPM in the treatment of persons with HTN and multimorbidity, including patients with obesity, CKD, diabetes, and CVDs, and has further emphasized the significance of the co-intervention intensity in relation to the treatment and management of specific comorbidities.

Consequently, regarding SBPM adherence in persons with HTN and multimorbidity, variable outcomes have been reported across studies [4,31]. Among the notable factors affecting patients’ adherence include the intricacies involved in the management of multimorbidity, the self-care burden, and cognitive decline, particularly in older patients. Approaches that incorporate SBPM with other interventions, including telemonitoring and various mobile health apps, have been found to enhance adherence among persons with HTN and multimorbidity [32,33]. Still, Stergiou et al. disclosed that persons with HTN and multimorbidity receiving feedback and reminders via mobile phone apps were increasingly consistent with regard to adherence to the SBPM protocols and instructions, taking their BP measurement on a daily basis [3,34]. Moreover, despite being a recommended aspect of SBPM success in the treatment and management of persons with HTN and multimorbidity, patient adherence often faces a number of significant challenges. For instance, the intricacies associated with the management of several chronic conditions, the self-care-associated burdens, and cognitive decline usually result in reduced patient adherence rates [35]. Several studies have also reported adherence difficulties, particularly in elderly persons with various types of cognitive impairments and disorders [27,33]. Such patients have been noted to experience difficulties in utilizing BP monitoring devices and providing precise interpretation of the reading. To resolve this challenge, simplified BP monitoring devices alongside educational interventions have been recommended as the most appropriate approach to enhancing adherence in such populations. The adherence challenges may also be enhanced through the use of co-interventions that make use of technology, including telemonitoring and mobile healthcare apps, which have indicated the ability to improve patient adherence [4,35]. The support, along with various educational programs developed by healthcare professionals, are important in ascertaining the appropriate usage of SBPM tools by persons with HTN and multimorbidity and ensuring comprehension of the importance of the readings. The simplification of SBPM tools and the provision of clear and understandable instructions are also likely to assist in enhancing patient adherence, particularly in elderly persons with HTN and those with cognitive impairments [36].

Regarding the integration of SBPM into the clinical practice contexts, it is noteworthy that a number of studies have indicated various challenges related to the integration of the intervention into regular clinical practices for persons with HTN and multimorbidity. Among the notable challenges is the absence of effective coordination between specialists tasked with the management and treatment of various chronic conditions and the primary care providers [37]. In certain instances, it has been noted that the obligation to interpret SBPM data and subsequently make decisions on treatment was imprecise and resulted in delays in medication and treatment adjustments [13]. To this end, various studies have recommended the usage of multidisciplinary care teams comprising healthcare professionals drawn from various fields, including primary care physicians, nurses, specialists, and pharmacies. Moreover, a recent study conducted by Cappuccio et al. disclosed that SBPM was found to be effective in instances where it formed part of the comprehensive care plan comprising routine follow-up, lifestyle counseling, and medication titration [9].

Furthermore, the successful incorporation of SBPM for persons with HTN and multimorbidity into the clinical practice calls for an increasingly coordinated approach that includes multidisciplinary healthcare professionals’ teams comprising specialists, pharmacists, nurses, primary care providers, and other healthcare experts [8,36]. Such teams are capable of not only collaborating to offer an effective reading of SBPM data but also ensuring effective adjustment of medications and treatment plans. Effective communication between the members of such teams is important to ascertain the provision of timely medical interventions to persons with HTN and multimorbidity, as per the SBPM data provided [8]. Still, EHR utilization alongside other notable digital tools is vital in sharing the SBPM data from the patient among the members of the healthcare team, given that this allows for an increasingly coordinated and effective care provision [24,36]. Nevertheless, the successful execution of such systems calls for increased investment in healthcare providers’ training and technology infrastructure.

## Conclusions

In conclusion, the evidence gathered in this systematic review has supported the usage of SBPM in the management of persons with HTN and multimorbidity, as it is an increasingly effective tool capable of significantly enhancing BP control in persons with HTN and multimorbidity. The use of SBPM not only enables persons with HTN to detect BP changes in real-time but also facilitates access to early interventions, which enables the prevention of potential complications, including kidney failure, stroke, and heart attack. This is a significant aspect, especially in persons with HTN and multimorbidity, who face a higher risk for adverse care outcomes resulting from the interaction between the various comorbid conditions. Nevertheless, SBPM’s efficacy is increasingly reliant on its effectual incorporation into clinical practice workflows and the adherence of the patients. In this regard, it is noteworthy that research that has integrated co-interventions like telemonitoring with SBPM and regular communication with healthcare professionals documented significant improvements in care outcomes compared to instances where SBPM was only used. The implication is that, in treating persons with HTN and multimorbidity, SBPM must not be employed as a standalone intervention but as a key component of the comprehensive care approach that takes support from healthcare providers and regular follow-ups. It is, therefore, recommended that prospective studies should concentrate on the evaluation of factors hindering the execution of SBPM, especially in underserved populations who bear the biggest burden of HTN and related comorbidities with regard to morbidity and mortality rates. Efforts should be made to increase access to validated SBPM devices and ensure that patients receive adequate training and support. Additionally, healthcare systems must develop standardized protocols for integrating SBPM data into clinical practice, including telemonitoring and EHR systems. Moreover, there is a need for additional studies focusing on the long-term effects and results of SBPM usage on persons with HTN and multimorbidity. Even as short-term studies have indicated enhancements in BP control and adherence among patients, the long-term effects of SBPM with regard to clinical outcomes, including mortality and cardiovascular events, have remained unclear.

## References

[1] Poblete JY, Vawter NL, Lewis SV (2023). Digitally based blood pressure self-monitoring program that promotes hypertension self-management and health education among patients with low-income: usability study. JMIR Hum Factors.

[2] Bray EP, Holder R, Mant J, McManus RJ (2010). Does self-monitoring reduce blood pressure? Meta-analysis with meta-regression of randomized controlled trials. Ann Med.

[3] Mulè G, Sorce A, Carollo C, Geraci G, Cottone S (2019). Self-blood pressure monitoring as a tool to increase hypertension awareness, adherence to antihypertensive therapy, and blood pressure control. J Clin Hypertens (Greenwich).

[4] De León-Robert A, Gascón-Cánovas JJ, Antón-Botella JJ, Hidalgo-García IM, López-Alegría C, Pérez-Cabrera YD, Campusano-Castellanos HM (2019). Validity of self blood pressure measurement in the control of the hypertensive patient: factors involved. BMC Cardiovasc Disord.

[5] Tran J, Norton R, Canoy D (2021). Multi-morbidity and blood pressure trajectories in hypertensive patients: a multiple landmark cohort study. PLoS Med.

[6] Muqadas K, Rahman MA, Akhlaq M (2024). Effectiveness of self-monitoring of blood pressure during multimorbidity; a review article. Chron Biomed Sci.

[7] Yang C, Lee DT, Wang X, Chair SY (2022). Effects of a nurse-led medication self-management intervention on medication adherence and health outcomes in older people with multimorbidity: a randomised controlled trial. Int J Nurs Stud.

[8] Whelton PK, Carey RM, Aronow WS (2018). 2017 ACC/AHA/AAPA/ABC/ACPM/AGS/APhA/ASH/ASPC/NMA/PCNA Guideline for the prevention, detection, evaluation, and management of high blood pressure in adults: A Report of the American College of Cardiology/American Heart Association Task Force on Clinical Practice Guidelines. Hypertension.

[9] Cappuccio FP, Kerry SM, Forbes L, Donald A (2004). Blood pressure control by home monitoring: meta-analysis of randomised trials. BMJ.

[10] Banegas JR, Graciani A, de la Cruz-Troca JJ (2012). Achievement of cardiometabolic goals in aware hypertensive patients in Spain: a nationwide population-based study. Hypertension.

[11] Gabb GM, Mangoni AA, Anderson CS (2016). Guideline for the diagnosis and management of hypertension in adults - 2016. Med J Aust.

[12] Morawski K, Ghazinouri R, Krumme A (2018). Association of a smartphone application with medication adherence and blood pressure control: the MedISAFE-BP Randomized Clinical Trial. JAMA Intern Med.

[13] Uhlig K, Patel K, Ip S, Kitsios GD, Balk EM (2013). Self-measured blood pressure monitoring in the management of hypertension: a systematic review and meta-analysis. Ann Intern Med.

[14] Bryant KB, Sheppard JP, Ruiz‐Negrón N (2020). Impact of self‐monitoring of blood pressure on processes of hypertension care and long‐term blood pressure control. J Am Heart Assoc.

[15] McManus RJ, Mant J, Bray EP (2010). Telemonitoring and self-management in the control of hypertension (TASMINH2): a randomised controlled trial. Lancet.

[16] Tinetti ME, Fried TR, Boyd CM (2012). Designing health care for the most common chronic condition - multimorbidity. JAMA.

[17] (2024). Hypertension prevalence among adults aged 18 and over: United States, 2017-2018. https://www.cdc.gov/nchs/products/databriefs/db364.htm.

[18] Wallace E, Salisbury C, Guthrie B, Lewis C, Fahey T, Smith SM (2015). Managing patients with multimorbidity in primary care. BMJ.

[19] Imai Y, Obara T, Asamaya K, Ohkubo T (2013). The reason why home blood pressure measurements are preferred over clinic or ambulatory blood pressure in Japan. Hypertens Res.

[20] Valderas JM, Starfield B, Sibbald B, Salisbury C, Roland M (2009). Defining comorbidity: implications for understanding health and health services. Ann Fam Med.

[21] Muntner P, McManus RJ, Shimbo D, de la Sierra A, Myers MG (2020). Home blood pressure monitoring: cost-effectiveness, patients’ preference and barriers for clinical use. Home Blood Press Monit.

[22] Grant RW, Pandiscio JC, Pajolek H, Woulfe A, Pelletier A, Kvedar J, Park ER (2012). Implementation of a web-based tool for patient medication self-management: the Medication Self-titration Evaluation Programme (Med-STEP) for blood pressure control. Inform Prim Care.

[23] Maher RL, Hanlon J, Hajjar ER (2014). Clinical consequences of polypharmacy in elderly. Expert Opin Drug Saf.

[24] McGinnis PB, Hunsberger M, Davis M, Smith J, Beamer BA, Hastings DD (2010). Transitioning from CHIP to CHIRP: blending community health development with community-based participatory research. Fam Community Health.

[25] Hatef E, Lans D, Bandeian S, Lasser EC, Goldsack J, Weiner JP (2022). Outcomes of in-person and telehealth encounters during COVID-19 within a large commercially insured cohort. JAMA Netw Open.

[26] Kaambwa B, Bryan S, Jowett S (2014). Telemonitoring and self-management in the control of hypertension (TASMINH2): a cost-effectiveness analysis. Eur J Prev Cardiol.

[27] Sheppard JP, Tucker KL, Davison WJ (2020). Self-monitoring of blood pressure in patients with hypertension-related multi-morbidity: systematic review and individual patient data meta-analysis. Am J Hypertens.

[28] Tucker KL, Sheppard JP, Stevens R (2015). Individual patient data meta-analysis of self-monitoring of blood pressure (BP-SMART): a protocol. BMJ Open.

[29] Katon WJ, Lin EH, Von Korff M (2010). Collaborative care for patients with depression and chronic illnesses. N Engl J Med.

[30] Kerry SM, Markus HS, Khong TK (2013). Home blood pressure monitoring with nurse-led telephone support among patients with hypertension and a history of stroke: a community-based randomized controlled trial. CMAJ.

[31] Allaham KK, Feyasa MB, Govender RD (2022). Medication adherence among patients with multimorbidity in the United Arab Emirates. Patient Prefer Adherence.

[32] Chun-Yun Kang G (2022). Technology-based interventions to improve adherence to antihypertensive medications - an evidence-based review. Digit Health.

[33] Volpi SS, Biduski D, Bellei EA, Tefili D, McCleary L, Alves AL, De Marchi AC (2021). Using a mobile health app to improve patients' adherence to hypertension treatment: a non-randomized clinical trial. PeerJ.

[34] Shimbo D, Artinian NT, Basile JN, Krakoff LR, Margolis KL, Rakotz MK, Wozniak G (2020). Self-measured blood pressure monitoring at home: a joint policy statement from the American Heart Association and American Medical Association. Circulation.

[35] Mills KT, Bundy JD, Kelly TN (2016). Global disparities of hypertension prevalence and control: a systematic analysis of population-based studies from 90 countries. Circulation.

[36] Zhao D, Liu J, Wang M, Zhang X, Zhou M (2019). Epidemiology of cardiovascular disease in China: current features and implications. Nat Rev Cardiol.

[37] Clark CE, Smith LF, Taylor RS, Campbell JL (2010). Nurse led interventions to improve control of blood pressure in people with hypertension: systematic review and meta-analysis. BMJ.

